# Plant volatile eliciting FACs in lepidopteran caterpillars, fruit flies, and crickets: a convergent evolution or phylogenetic inheritance?

**DOI:** 10.3389/fphys.2014.00121

**Published:** 2014-04-01

**Authors:** Naoko Yoshinaga, Hiroaki Abe, Sayo Morita, Tetsuya Yoshida, Takako Aboshi, Masao Fukui, James H. Tumlinson, Naoki Mori

**Affiliations:** ^1^Division of Applied Life Sciences, Graduate School of Agriculture, Kyoto UniversitySakyo, Kyoto, Japan; ^2^Division of Applied Biosciences, Graduate School of Agriculture, Kyoto UniversitySakyo, Kyoto, Japan; ^3^Department of Entomology, Center for Chemical Ecology, Pennsylvania State UniversityUniversity Park, PA, USA

**Keywords:** Lepidoptera, *Drosophila melanogaster*, *Teleogryllus*, Amino acids, Deamination, FACs

## Abstract

Fatty acid amino acid conjugates (FACs), first identified in lepidopteran caterpillar spit as elicitors of plant volatile emission, also have been reported as major components in gut tracts of *Drosophila melanogaster* and cricket *Teleogryllus taiwanemma*. The profile of FAC analogs in these two insects was similar to that of tobacco hornworm *Manduca sexta*, showing glutamic acid conjugates predominantly over glutamine conjugates. The physiological function of FACs is presumably to enhance nitrogen assimilation in *Spodoptera litura* larvae, but in other insects it is totally unknown. Whether these insects share a common synthetic mechanism of FACs is also unclear. In this study, the biosynthesis of FACs was examined *in vitro* in five lepidopteran species (*M. sexta*, *Cephonodes hylas*, silkworm, *S. litura*, and *Mythimna separata*), fruit fly larvae and *T. taiwanemma*. The fresh midgut tissues of all of the tested insects showed the ability to synthesize glutamine conjugates *in vitro* when incubated with glutamine and sodium linolenate. Such direct conjugation was also observed for glutamic acid conjugates in all the insects but the product amount was very small and did not reflect the *in vivo* FAC patterns in each species. In fruit fly larvae, the predominance of glutamic acid conjugates could be explained by a shortage of substrate glutamine in midgut tissues, and in *M. sexta*, a rapid hydrolysis of glutamine conjugates has been reported. In crickets, we found an additional unique biosynthetic pathway for glutamic acid conjugates. *T. taiwanemma* converted glutamine conjugates to glutamic acid conjugates by deaminating the side chain of the glutamine moiety. Considering these findings together with previous results, a possibility that FACs in these insects are results of convergent evolution cannot be ruled out, but it is more likely that the ancestral insects had the glutamine conjugates and crickets and other insects developed glutamic acid conjugates in a different way.

## Introduction

Many plants respond to herbivory by an induced release of volatile organic compounds (VOCs), which are important chemical cues for natural enemies of the herbivores (Turlings et al., 1990; Kessler and Baldwin, 2001). Numerous studies have shown that this ingenious plant defense system is triggered by substances in the regurgitants of the herbivores. The best known of these plant volatile elicitors are the fatty acid amino acid conjugates (FACs) that first were identified from beet armyworm, *Spodoptera exigua*, larvae (Alborn et al., 1997) but later also found in several other lepidopteran species (Pohnert et al., 1999; Halitschke et al., 2001; Mori et al., 2001, 2003; Alborn et al., 2003; De Moraes and Mescher, 2004; Yoshinaga et al., 2010; Mori and Yoshinaga, 2011). Of the FACs, volicitin [*N*-(17-hydroxylinolenoyl)-L-glutamine], is the most active elicitor for seedlings of *Zea mays* cultivars (Alborn et al., 1997, 2003; Sawada et al., 2006). Of the other FACs often found in lepidopteran larvae, *N*-linolenoyl-L-glutamine is active in *Z. mays* and in several other species of plants (Schmelz et al., 2009). FACs with negligible activity are glutamine conjugates with linoleic, oleic, and other minor fatty acids (Pohnert et al., 1999; Turlings et al., 2000; Mori et al., 2003).

**Figure 1:**
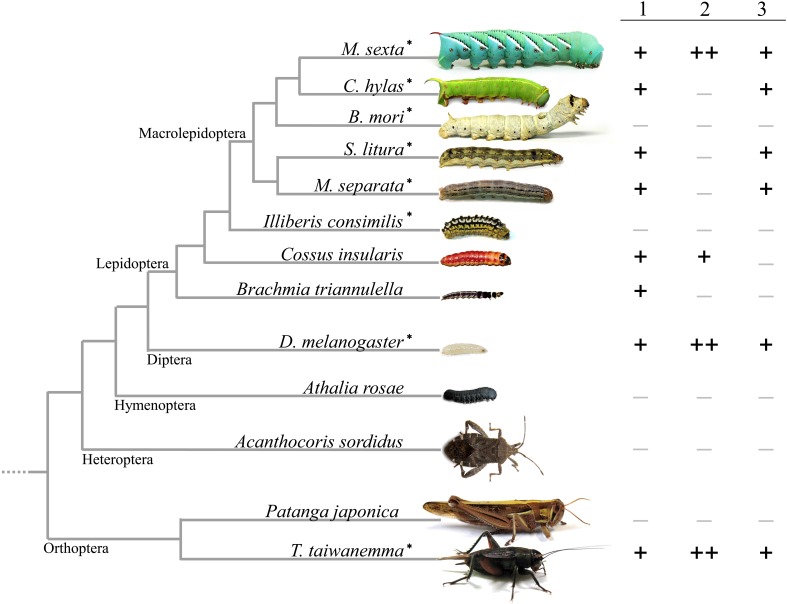
**Overview of FAC analog patterns in insects**. 1, glutamine conjugates; 2, glutamic acid conjugates; 3, hydroxylated FACs. All FAC-containing species had glutamine conjugates (1) in common, and the evolutionarily earliest species in Lepidoptera had only this type of FACs, suggesting this might be the evolutionarily older FACs. ++: major components, +: components constantly detected, −: not detected. *: insect species used in this study.

Screening FACs of 29 lepidopteran species, we found them in 19 of these species, suggesting FACs are commonly synthesized through a broad range of lepidopteran caterpillars (Yoshinaga et al., 2010). Figure 1 shows the summary of FAC patterns in insect species so far examined. Since all FAC-containing species had glutamine conjugates [Figure 1, (1) in common, and the evolutionarily earliest species *Brachmia triannulella* had only this type of FAC (*N*-linolenoyl-L-glutamine and *N*-linoleoyl-L-glutamine), the glutamine conjugates might be the evolutionarily older FACs. Furthermore, some species had glutamic acid conjugates (2), and some had hydroxylated FACs (3)]. Comparing the diversity of FACs with lepidopteran phylogeny indicates that glutamic acid conjugates can be synthesized by relatively primitive species (*Cossus insularis*), while hydroxylation of fatty acids is limited mostly to larger and more developed macrolepidopteran species. FACs were also found in other insects. We screened 13 non-lepidopteran insects for the presence of FACs and found these compounds present in adults of two closely related cricket species *Teleogryllus taiwanemma* and *T. emma* (Orthoptera: Gryllidae), and the fruit fly, *Drosophila melanogaster* (Diptera: Drosophilidae) (Yoshinaga et al., 2007). The FAC analogs in these insects were again glutamine-/glutamic acid-conjugates with hydroxylated FACs, and no other new FACs were detected (Figure 1). Also some katydids are reported to have FACs, but the detail data are not shown (Alborn et al., 2007).

In a previous study, we showed that *N*-linolenoyl-L-glutamine in larval *S. litura* plays an important role in nitrogen assimilation which might be an explanation for caterpillars synthesizing FACs despite an increased risk of attracting natural enemies (Yoshinaga et al., 2008). Whether the FACs in other insects (crickets, fruit fly, and some lepidopteran species) function in the same manner or not is a question to be examined. At this point, their localization to the alimentary tract and the 3 rigid analogs (glutamine conjugates, glutamic acid conjugates, and hydroxylated FACs) are characteristic of FACs in the insects. These characteristics might be defined by the FAC synthesis in each species, but the biosynthetic mechanism is not fully understood in most insects. In lepidopteran species, the fatty acid moiety of the FAC molecule originates from the diet of the caterpillar (Paré et al., 1998). Consequently, the fatty acid composition of the FACs roughly reflects the fatty acid composition in the host plant, although there seems to be a preference for linolenic and linoleic acid in the FAC synthesis (Aboshi et al., 2007). But the amino acid moiety does not reflect the amino acid compositions in a diet nor that in alimentary tracts. *In vitro* biosynthesis showed the homogenates of fresh larval gut tissues conjugated glutamine with sodium linolenate. However, the same experiments using glutamic acid did not give the same results (Yoshinaga et al., 2005).

In this paper we examined *in vitro* biosynthesis of FACs with fresh gut tissues of lepidopteran caterpillars (*M. sexta*, *Cephonodes hylas*, *Bombyx mori*, *S. litura*, and *Mythimna separata*), *T. taiwanemma* and *D. melanogaster* larval bodies to figure out whether these insects share the same synthetic mechanism that could be inherited from a common ancestor, or each species developed FACs by its own pathway. The amino acid analysis of selected insect guts and the feeding experiment with adult crickets were further conducted to discuss the findings from different aspects.

## Materials and methods

### Insect rearing

Colonies of common cutworm, *S. litura*, were supplied by Ishihara Sangyo (Osaka, Japan) and eggs of oriental armyworm, *Mythimna separata*, were kindly supplied by Professor Yasuhisa Kunimi (Tokyo University of Agriculture and technology). Commercially available silkworm, *Bombyx mori*, eggs were purchased from Kougensya (Nagano, Japan) and these colonies were successively maintained. Tobacco hornworm, *Manduca sexta*, eggs were purchased from Carolina Biological supply Company (USA) and imported with permission of Japanese plant protection and fish and USA wildlife protection inspection. Oriental bee hawks, *Cephonodes hylas*, were collected in Kyoto. *Spodoptera litura* and *M. separata* were reared on artificial diet Insecta-LFS, and *B. mori* were on SilkMate 2S (Nihon Nosan Kogyo Ltd., Yokohama, Japan). *Manduca sexta* were reared on artificial diet (Southland products Inc., diet type: tobacco hornworm). Laboratory strains of *D. melanogaster* (Canton S, Oregon R) were reared on artificial diet Formula 4–24 (Carolina Biological supply Co.) added with dry yeast. *T. taiwanemma* crickets (Orthoptera: Gryllidae) were obtained in the field and reared on commercial diets (MF®, Oriental Yeast Co. Ltd., Tokyo), *ad libitum* at 25 ± 2°C, 60 ± 10% r.h. under a 16/8 h (light/dark) cycle. Newly emerged adults were removed every day from cultures containing numerous nymphs. Field-collected *Illiberis consimilis* and *Atrophaneura alcinous* larvae were fed with natural diet for a few days.

### Preparation of crude enzyme extracts

Last instars of lepidopteran caterpillars and adult crickets were anesthetized by immersion in crushed ice water for 5 min. Midgut tissue was dissected out and the contents were washed off then rinsed thoroughly with distilled water. The fresh tissue was homogenized with a 25 mM imidazole-HCl buffer (250 μl/50 mg gut tissue, pH 7.5, 0°C), and after centrifugation (15,000 g, 4°C, 5 min), the supernatant was used as crude enzyme extract. In the case of *D. melanogaster*, a preparatory experiment revealed FACs were detected from isolated midgut tracts but not the rest of body. So, for a reason of expediency, we used whole bodies instead of midgut tissues in this experiment. The whole bodies of last instar larvae were homogenized with a 25 mM imidazole-HCl buffer (50 μl/25 larvae, pH 7.5, 0°C) and prepared as described.

### *In vitro* biosynthesis of FACs with enzyme extracts

Fifty μl of crude enzyme extract was incubated with 12.5 μl of 50 mM sodium linoleate and 12.5 μl of 100 mM aqueous solution of glutamine (or ^15^N-glutamine for *M. sexta* case, to distinguish the products from remnant FACs) or glutamic acid (or ^15^N-glutamic acid for *M. sexta* and *D. melanogaster* case). The ^15^N-labeled substrates were used in some cases where the residual FACs in the enzyme extract interfered with the assay results. After incubation for 30 min at 30°C, all assay mixtures were boiled for 20 min to stop the enzymatic reactions and 25 μ l of acetonitrile containing 250 ng of *N*-palmitoleoyl-L-glutamine added as an internal standard. The mixtures were centrifuged for 5 min at 15,000 g and the supernatants filtrated by using a syringe filter (0.45 μm, DISMIC-13HP, Toyo Roshi Kaisya, Ltd). Control assay was conducted with 25 μl of distilled water instead of the substrates and net amount of product FACs was corrected by subtracting the residual FACs detected in the control sample.

### LCMS and LCMSMS analyses

Mass spectral measurements were carried out with an LCMS-2020 instrument (Shimadzu, Kyoto, Japan) combined with an HPLC system (LC-20AD pump, SIL-20ACHT system controller, and CTO-20A column oven, Shimadzu). A portion (5 μ l) of sample solution was injected into a reversed-phase column (Mightysil RP-18 GP 50 × 2.0 mm I.D., Kanto chemical Co., Inc.), eluted for 12 min at (0.2 ml/min) with a solvent gradient of 42–75% CH_3_CN containing 0.08% acetic acid, in water containing 0.05% acetic acid. The column temperature was maintained at 40°C (CTO-20A column oven, Shimadzu), and the column eluent was monitored by continuous MS total ion current trace. The CDL temperature was 250°C, the voltage was 1.5 kV, the nebulizer gas flow was 1.5 l/min, and the analytical mode was ESI negative scan from m/z 250–500. The negative ionization mass spectra gave characteristic [M–H]^−^ ions for *N*-linoleoyl-L-glutamine at m/z 407 (m/z 408 for ^15^N-labeled compounds), and *N*-linoleoyl-L-glutamic acid at m/z 408 (m/z 409 for ^15^N-labeled compounds).

Samples were also analyzed using the Prominence HPLC system coupled to LCMS-IT-TOF (Shimadzu, Kyoto) for LCMSMS analysis. Using the same HPLC conditions, the MS was operated at a probe voltage of 4.50 kV, CDL temperature of 200°C, block heater temperature of 200°C, nebulizer gas flow of 1.5 l/min, ion accumulation time of 30 ms, and the analytical mode was ESI-negative, SCAN.

### Synthesis of ^15^N-labeled FACs and incubation experiments

*N*-Linolenoyl-L-[α−^15^N] glutamine and *N*-linolenoyl-L-[γ−^15^N] glutamine were synthesized as described previously (Koch et al., 1999). The chemical structures of these synthesized compounds were confirmed by ^1^H-NMR (Sawada et al., 2006) and ^15^N-NMR (Yoshinaga et al., 2008) spectroscopy. To examine an *in vitro* conversion of glutamine conjugate to the glutamic acid conjugate, 10 μg of the synthesized *N*-linolenoyl-L-[α−^15^N] glutamine and *N*-linolenoyl-L-[γ−^15^N] glutamine were used as substrates. The fresh midgut tissue (50 mg) from adult crickets, homogenized with a 250 μl of 25 mM imidazole-HCl buffer (pH 7.5, 0°C), were mixed with substrates and incubated for 16 h at 4°C. A 100 μl solution of acetonitrile containing 1 μg of *N*-palmitoleoyl-L-glutamine (internal standard) was added to the assay mixture. It was centrifuged and the supernatant was filtrated for LCMS analysis as described above. Instead of a fresh midgut homogenate, a portion of the boiled tissue homogenate was used as a control.

### Feeding experiment with *N*-linolenoyl-L-[α-^15^N] glutamine in adult crickets

A cubic cm of artificial diet (Southland products Inc., diet type: tobacco hornworm) containing 1 μg of *N*-linolenoyl-L-[α−^15^N] glutamine was served to an adult cricket. Two hours after the diet was completely eaten, the cricket was anesthetized by immersion in crushed ice water and dissected to remove the alimentary tract. The tissue including content was homogenized with 300 μl of acetonitrile-water (1/1, v/v), and centrifuged as described above to obtain a supernatant for LCMS analysis.

### Amino acid analyses

Fresh midgut tissues were dissected out from *S. litura*, and *M. sexta* larvae as described above. A tungsten knife was used to dissect out midgut tissues from last instar of *D. melanogaster* larvae. The gut tissues rinsed thoroughly by distilled water were homogenized in a portion of 2 N HCl aq. containing 5 μl of β-alanine solution (1 mg/ml) as internal standard, and roughly centrifuged to get the supernatant. The amino acid extracts were loaded on a cation exchange cartridge Oasis MCX (6 ml; Waters) and extracted with 4 N ammonia solution (50% water/methanol). After evaporation and dilution in 100 μl of water, the amino acid sample was mixed with 80 μl of ethanol/pyridine (4/1) mixture and 10 μl of ethyl chloroformate and gently shaken for 5 min to derivatize each amino acid by the method of Silva et al. (2003). The reaction solution was extracted with 200 μl of dichloromethane. Then, aliquots (1.0 μl) of each sample were analyzed by gas chromatography-mass spectrometry (GCMS) (HP-5890 plus series II gas chromatograph with a 30 m × 0.32 mm, 0.33-μm film thickness, HP-5MS capillary column, interfaced to an HP-5989B mass spectrometer; Hewlett-Packard). The column temperature was held at 100°C for 5 min after injection and then programmed at 10°C/min to 290°C.

For quantitative analyses of amino acids, the samples were analyzed by GC (HP-6850 gas chromatograph with a 25 m × 0.2 mm, 0.33-μm film thickness, HP-5 capillary column; Hewlett-Packard) under the same analytical conditions as those used for GCMS analysis.

## Results

**Figure 2:**
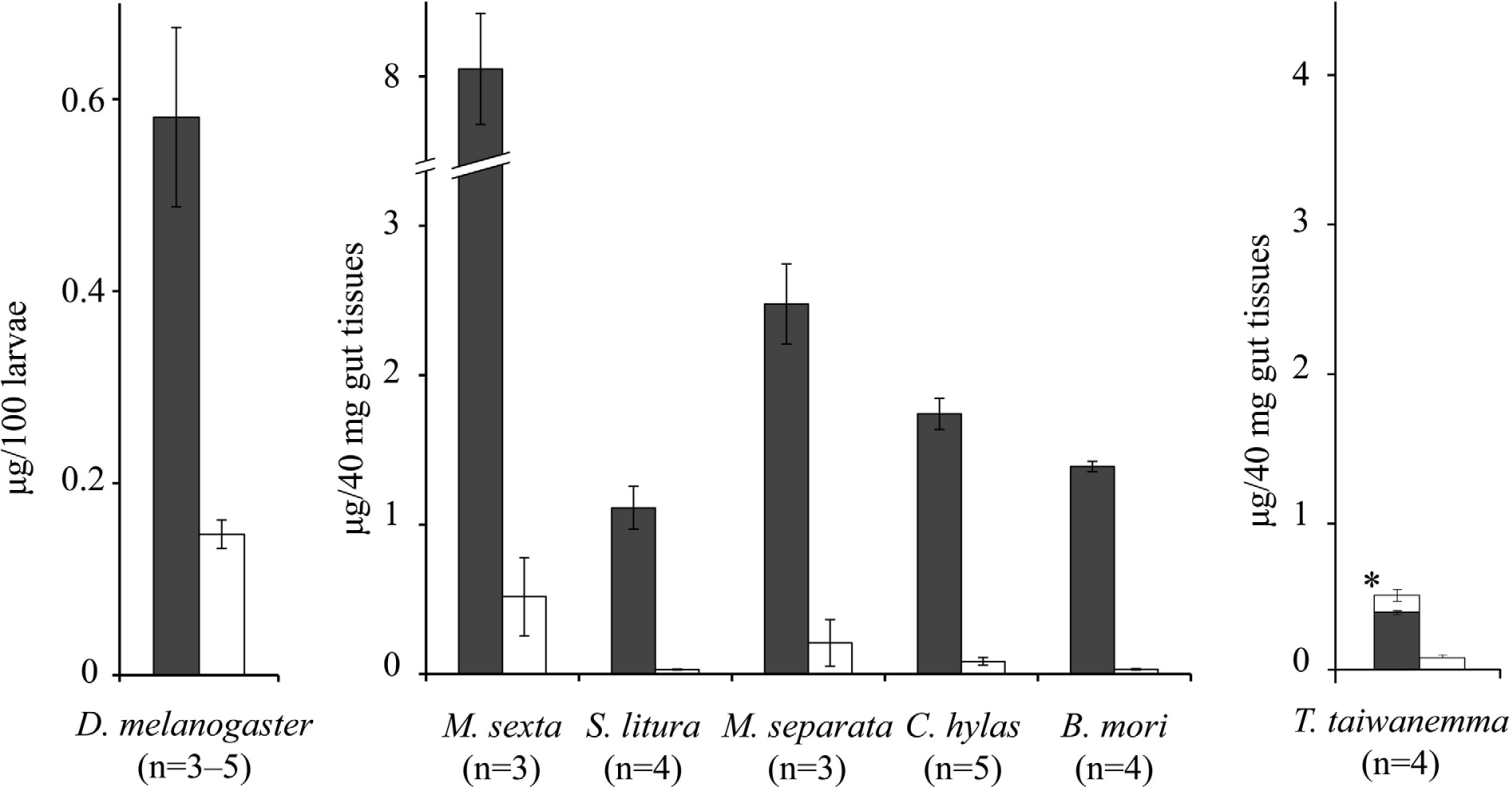
**The amounts of FACs synthesized *in vitro* (Mean ± s.e.m.)**. The amounts of conjugates synthesized by fresh midgut tissues of Lepidoptera species and *Teleogryllus* crickets, or whole *Drosophila* larval body homogenized and incubated with sodium linoleate and glutamine (left) or glutamic acid (right). Solid bars: product *N*-linoleoyl-L-glutamine, open bars: product *N*-linoleoyl-L-glutamic acid (^*^converted from *N*-linoleoyl-L-glutamine).

In a previous study, we reported that fresh midgut tissue from *S. litura* larvae did not synthesize glutamic acid conjugates, when incubated with glutamic acid and sodium linolenate (Yoshinaga et al., 2005). Subsequently, however, a sensitive Shimadzu LCMS2020 using SIM mode enabled us to detect a trace amount of glutamic acid conjugates synthesized in the assay. As shown in Figure 2, it was synthesized by the same method as glutamine type FACs, but the amount synthesized was much less. In the case of *D. melanogaster*, the amount was less than 30% of that of glutamine type FACs. Such strong selection for glutamine was commonly observed in 5 lepidopteran species examined here. It was not surprising that *M. sexta* midgut tissues had an ability of synthesizing glutamic acid conjugates, but the same pattern was seen in other lepidopteran species which do not have glutamic acid type FACs in nature. Furthermore, the FACs synthesized *in vitro* by silkworm gut tissue was totally unexpected, because we have never found FACs in living silkworm midgut or regurgitant, regardless of diet conditions. The same experiments using two other lepidopteran species, *Illiberis consimilis* and *Atrophaneura alcinous*, larvae which do not have FACs, did not show any conjugate products (data not shown). Although a similar pattern was observed in *Teleogryllus* crickets assay, a crucial difference was additional production of the glutamic acid conjugates when incubated with glutamine (Figure 2, *T. taiwanemma*, left bar), which was not observed in other insect cases. Further extension of the incubation period increased not only the total FAC production but also the ratio of glutamic acid conjugates against glutamine conjugates, suggesting a conversion to glutamic acid conjugates occurred in parallel.

**Figure 3:**
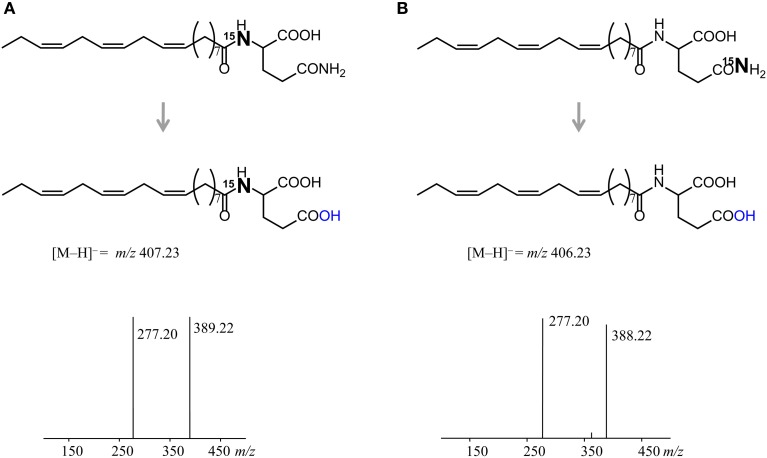
**Structures and MS^2^ spectra of product *N*-linolenoyl-L-glutamic acid**. *N*-Linolenoyl-L-[α−^15^N] glutamine was converted to^15^N-labeled *N*-linolenoyl-L-[α−^15^N] glutamic acid **(A)** and *N*-linolenoyl-L-[γ−^15^N] glutamine yielded unlabeled *N*-linolenoyl-L-glutamic acid **(B)**. ^15^N-labeling at the α-position of the glutamine moiety was passed to corresponding glutamic acid conjugate, whereas ^15^N-labeling at the γ-position (side chain of glutamine) was cleaved off during its conversion.

To verify the *in vitro* conversion of FACs, a similar incubation assay was conducted for crickets using synthesized *N*-linolenoyl-L-[α−^15^N] glutamine or *N*-linolenoyl-L-[γ−^15^N] glutamine as substrates. In both cases a distinct peak of *N*-linolenoyl-L-glutamic acid was observed but its [M–H]^−^ ion was different. The *N*-linolenoyl-L-glutamic acid made from *N*-linolenoyl-L-[α−^15^N] glutamine gave [M–H]^−^ ion at m/z 407.23, indicating presence of ^15^N-label (Figure 3A), while the other gave it at m/z 406.23 (no label, B). The dehydrated daughter ion was observed at m/z 389.22 (Figure 3A) and 388.22 (Figure 3B) together with linolenic acid ion at m/z 277.20, as previously reported (Yoshinaga et al., 2007). The results clearly showed that ^15^N-labeling at the α-position of the glutamine moiety was passed to corresponding glutamic acid conjugate (A), whereas ^15^N-labeling at the γ-position (side chain of glutamine) was cleaved off during its conversion (B). Using fruit flies and lepidopteran caterpillars resulted in rapid degradation of the substrate glutamine conjugates. The conversion of FACs was further examined in living crickets by a feeding experiment using *N*-linolenoyl-L-[α−^15^N] glutamine. Two hours of feeding FACs was enough to detect *N*-linolenoyl-L-[α−^15^N] glutamic acid in the gut contents. The percentage of glutamine-FACs converted was calculated to be 48.6 ± 17.0 % (mean ± s.e.m., *n* = 4). Hydroxylation to yield volicitin was not observed.

**Figure 4:**
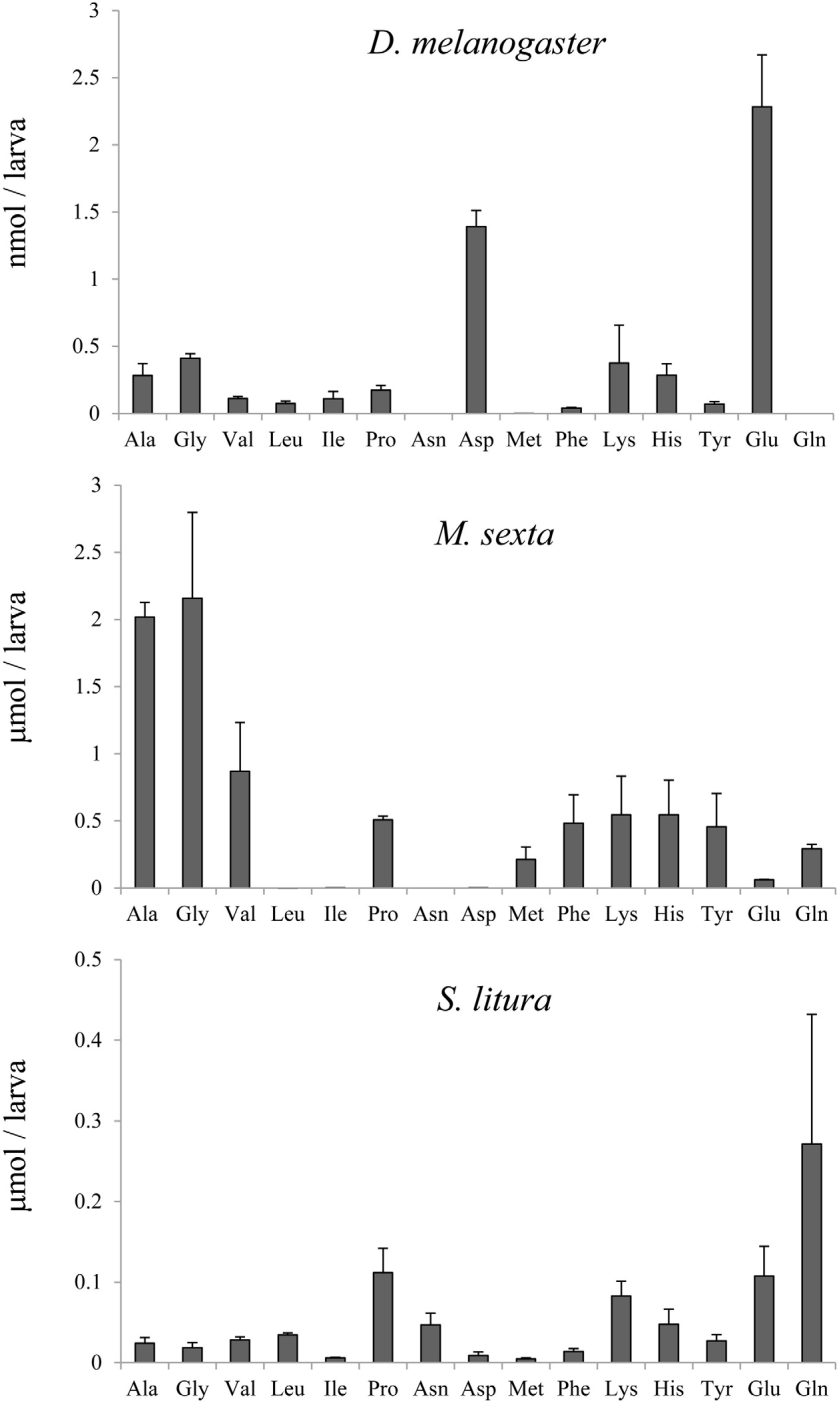
**Amino acid compositions in larval midgut tissues of *D. melanogaster*, *M. sexta* and *S. litura* (*n* = 3, Mean ± s.e.m.)**.

Amino acid composition in the midgut tissues of *D. melanogaster*, *M. sexta*, and *S. litura* were analyzed by GC and GCMS (Figure 4). The *S. litura* data was not identical to the same experimental data in our previous paper (Yoshinaga et al., 2008), probably because of the difference of insect colonies and/or dietary lot quality, but the ratio of glutamine/glutamic acid was consistently reproduced. Glutamine predominated over glutamic acid. In fruit flies, the most notable difference from *S. litura* and *M. sexta* data was the glutamine/glutamic acid ratio. Glutamic acid was the predominant amino acid while glutamine was below the limit of detection.

## Discussion

It was known that glutamine-type FACs are easily synthesized *in vitro* by lepidopteran midgut tissues incubated with free glutamine and fatty acid substrates. However, the same experiment using glutamic acid as substrate did not clearly give a product conjugate (Lait et al., 2003; Yoshinaga et al., 2005). We thought it could be caused by a lack of cofactor in the assay, or the biosynthetic pathway of glutamic acid conjugates might be completely different from that of glutamine conjugates. But our results here showed fresh midgut tissues of all of the tested insects have an ability to synthesize glutamic acid conjugates *in vitro* when incubated with free glutamic acid.

**Figure 5:**
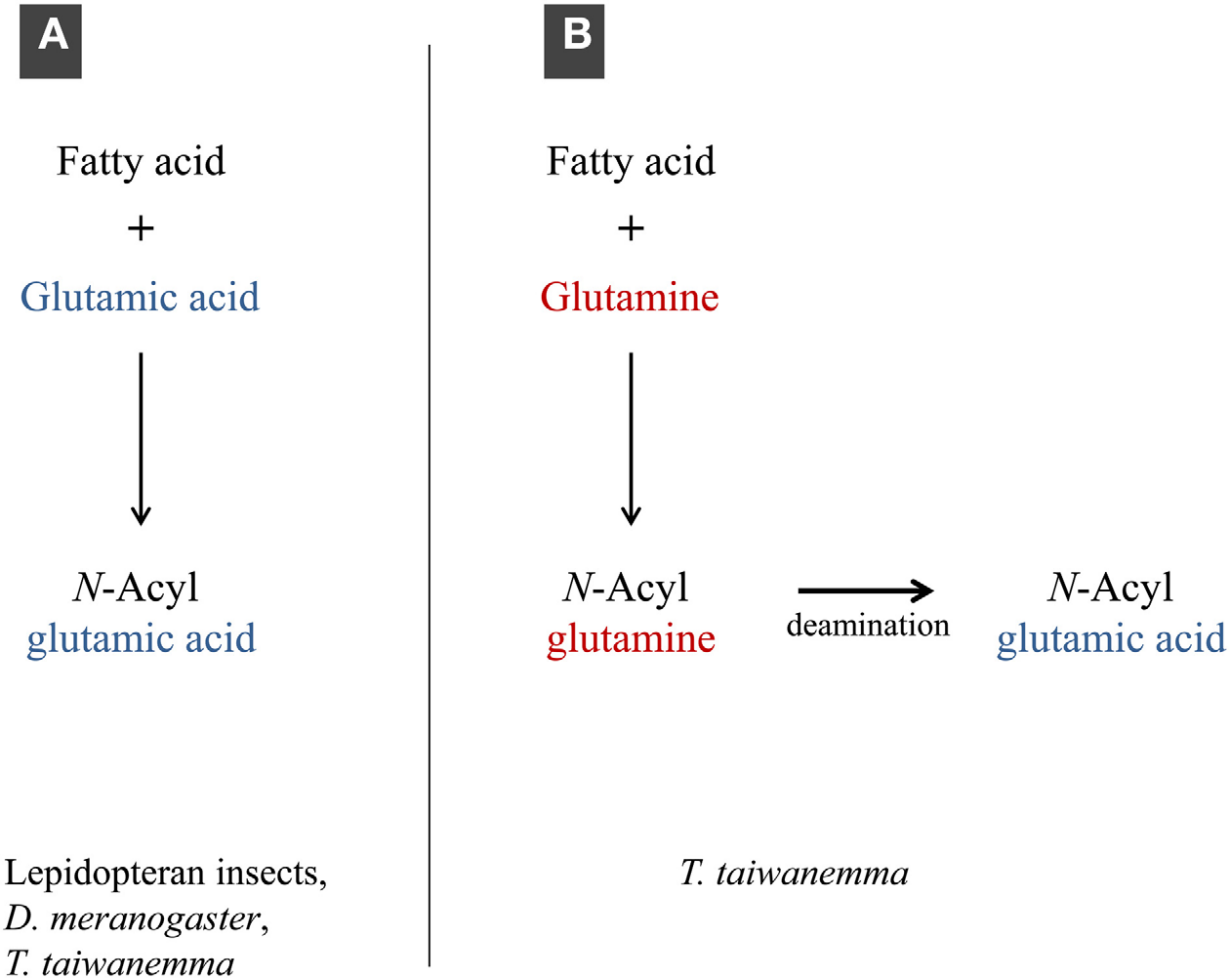
**Two biosynthetic pathways of glutamic acid conjugates. (A)**, direct conjugation observed in lepidopteran insects, *D. melanogaster* and *Teleogryllus* crickets; **(B)**, deamination in *Teleogryllus* crickets.

The total amount of conjugated product in the cricket case was relatively small compared to other insects but it shows a unique pathway of synthesizing glutamic acid conjugates in addition to the direct conjugation which is common to other insects (Figure 5). The deamination was clearly verified by the subsequent experiment using ^15^N-labeled FACs and also reproduced *in vivo* by the feeding experiments using adult crickets. It is not clear which could be the main pathway for the biosynthesis of glutamic acid conjugates in crickets, but we assume both direct conjugation and deamination together will explain the predominance of the glutamic acid conjugates *in vivo* in this cricket species.

In other insects, the *in vitro* FAC synthesis did not replicate the *in vivo* FAC pattern. Although fruit fly and *M. sexta* larvae have predominant glutamic acid conjugates, and silkworms have no FACs, the *in vitro* assay results denoted a constant tendency of effective synthesis of glutamine conjugates but only a small amount of glutamic acid conjugates (Figure 2). The possibility that these products might be artifacts can be refuted by preliminary results with two other species, *I. consimilis* and *A. alcinous* (data not shown). These species do not have FACs and their midgut tissues did not show any detectable FAC synthetic ability. Therefore, silkworm midgut tissues seem to contain a FAC conjugase just like other species, but for an unknown reason they do not synthesize FACs *in vivo*. What could be the factor determining the FAC pattern in insects? A direct factor could be the amino acid availability in the tissues. In fruit flies, free glutamic acid was dominant in the tissues and can be the prior substrate for FACs, because free glutamine was negligible (Figure 3). *Manduca sexta* larval midgut tissues contained free glutamine, that enable the larvae to synthesize corresponding amounts of glutamine conjugates. Alborn et al. (2003) reported that glutamine conjugates in *M. sexta* were degraded quickly while glutamic acid conjugates were stable at room temperature, suggesting the hydrolytic selectivity affects the FAC pattern in this species. However, the silkworm case cannot be explained by the hydrolysis. The hydrolysis assay using silkworm and *S. litura* gut contents incubated with *N*-palmitoleoyl-L-glutamine (artificial FACs) showed the degradation speed in silkworm was slower than *S. litura* (data not shown). Further study is necessary to explain the absence of FACs in living silkworm larvae.

Since the first identification of FACs from outside of lepidopteran insects, whether the FACs in these insects is a result of convergent evolution or a phylogenetic inheritance from an ancestral insect has been controversial (Yoshinaga et al., 2007). In our previous study about lepidopteran FACs, we hypothesized that glutamine conjugates are the evolutionarily older FACs and the other two type of analogs (glutamic acid conjugates and hydroxylated FACs) might be recently derived (Yoshinaga et al., 2010). However, the idea was seemingly contradictory to the fact that fruit flies and *Teleogryllus* crickets have the same profile of FAC analogs with *M. sexta*, which is an evolutionally-advanced lepidopteran species (Figure 1). The results in this study were consistent with either previous result. The FAC conjugase from the insects tested here showed similar ability of direct coupling of glutamine/glutamic acid with fatty acid. And the predominance of glutamic acid conjugates in crickets, *D. melanogaster* and *M. sexta* were differently explained. A glutamine deficiency in *D. melanogaster* midgut tissues and a rapid hydrolysis of glutamine conjugates in *M. sexta* may not be conclusive and the possibility of another explanation in both cases remains, but the deamination was clearly unique to the crickets. If these insects have developed their own pathway to make glutamic acid conjugates for a physiological necessity, it is interesting that these compounds are synthesized in the same organ, but through different pathways in different species. The function and genetics of FAC biosynthesis in these insects will be discussed in a future study.

### Conflict of interest statement

The authors declare that the research was conducted in the absence of any commercial or financial relationships that could be construed as a potential conflict of interest.
